# *Chenopodium ambrosioides* as a bone graft substitute in rabbits radius fracture

**DOI:** 10.1186/s12906-017-1862-5

**Published:** 2017-07-04

**Authors:** Vicente F. Pinheiro Neto, Rachel M. Ribeiro, Camila S. Morais, Matheus B. Campos, Denilson A. Vieira, Porfírio C. Guerra, Ana L. Abreu-Silva, José R. Silva Junior, Flavia Raquel F. Nascimento, Marilene O. R. Borges, Antonio C.R. Borges

**Affiliations:** 1 Departamento de Medicina, Laboratório de Cirurgia Experimental, Centro Universitário do Maranhão, São Luís, Maranhão Brazil; 2 Departamento de Ciências Fisiológicas, Laboratório de Farmacologia, Universidade Federal do Maranhão, Av. dos Portugueses, 1966 - Bacanga, São Luis, MA 65080-805 Brazil; 3 Departamento de Medicina Veterinária, Universidade Estadual do Maranhão, São Luis, Maranhão Brazil; 4 Departamento de Patologia, Laboratório de Imunofisiologia, Universidade Federal do Maranhão, São Luís, Maranhão Brazil

**Keywords:** *Chenopodium ambrosioides*, Plant biomaterials, Heterograft, Bone regeneration, Fracture healing

## Abstract

**Background:**

Bone defects caused by trauma, infection or tumor resection are common in orthopedic clinic and depending on the extent of the fracture; the vast majority require treatment with bone substitutes. Among the bone grafts employees, the autograft is defined as the gold standard, but with some limitations, principally related to morbidity at the site of its removal, with the need to search for other biomaterials as adjuvant in bone regeneration. Therefore, the objective of the present study was to evaluate the use of *Chenopodium ambrosioides* as a bone graft substitute for the osseointegration of fractures in rabbits, compared to other bone grafts already employed in the surgical routine as *Ricinus communis* (castor oil) polyurethane and autogenous bone marrow.

**Methods:**

Forty-eight rabbits were divided into four groups. After anesthesia, a radius fracture was created and the animals were treated with the following grafts: *C. ambrosioides* graft, autogenous bone marrow, castor oil and saline (control). After 30, 60 and 90 days, the animals were submitted to radiographical and histological analyses and bone alkaline phosphatase, osteocalcin, biomechanical tension, and collagen were measured. We also realized a phytochemical screening and in vitro antioxidant activity including 1,1-Diphenyl-2-picrylhydrazyl (DPPH) radical.

**Results:**

The data showed that the growth of the bone callus was more expressive and biomechanical assessment showed better tensile strength in *C. ambrosioides* graft. The experimental results also revealed that there was significantly greater activity of the bone alkaline phosphatase and osteocalcin during early fracture healing, similarly to the group receiving autogenous bone marrow. Histologically, immature bone was observed in *C. ambrosioides* graft at 30 days, whereas the formation of cartilaginous tissue predominated in the other groups. A higher amount of type I collagen was observed in *C. ambrosioides* graft throughout treatment. It was detected strong presence of flavonoids and appreciable antioxidant activity.

**Conclusions:**

The results indicate that *C. ambrosioides* graft and autogenous bone marrow have similar ability to enhance bone regeneration, higher than the castor oil graft, suggesting that the medicinal plant can provide therapeutic benefits for bone tissue.

## Background

Bone grafting consists of the transplantation of biocompatible material at the site where significant loss of bone substance had occurred, in order to reconstruct bone defects generally caused by trauma, infection or tumor resection [1, 2].

Autologous bone or autograft represents up to this date, the gold standard and most effective method for bone regeneration as it promotes bone formation over its surface by direct bone bonding (osteoconduction) and induces local stem cells to differentiate into bone cells (osteoinduction) without any associated immune response. However, often results in a high donor site morbidity and its availability is limited [3–5].

The emergence of new bone graft options has generated significant uncertainties regarding the determination of the most adequate product for surgical procedures that use synthetic materials. Natural bone substitutes have been investigated including tissues obtained from the same species (allografts) or from different species (heterografts). These graft materials should be biocompatible and non-toxic and should not stimulate inflammatory processes [6, 7].

Use of medicinal plants for graft production is a promising alternative since they are biocompatible, easily applied and stored, and have been shown to favor bone growth [8]. *Chenopodium ambrosioides* L. (*syn. Dysphania ambrosioides* (L.) Mosyakin & Clemants), Chenopodiaceae, popularly known as “mastruz” or “erva-de-santa-maria”, is used by the population in Brazil and Latin America [9] as teas, infusions or syrups for the treatment of inflammatory conditions [10] and or contusions and fractures [11, 12]. In addition, *Chenopodium ambrosioides* occupies the 17th position on the National Register of Plants of Interest to the National Health System (Relação Nacional de Plantas de Interesse do Sistema Único de Saúde - RENISUS), a list compiled by the Brazilian Government which consists of 71 plant species used in folk medicine for the alternative treatment of health conditions [13]. Experimental studies have shown that *C. ambrosioides* exerts immunostimulatory [14, 15], antimicrobial [16], anti-inflammatory and antinociceptive activities [17, 18].

Studies from our group evaluating the effect of a poultice prepared from the leaves of *C. ambrosioides* on bone healing in rabbits have shown that this medicinal plant accelerates bone regeneration as demonstrated by radiological and histological analysis, highlighting the importance of medicinal plants as biomaterials [8]. Therefore, the objective of the present study was to evaluate the use of *C. ambrosioides* as a bone graft substitute for the osseointegration of fractures in rabbits, compared to other bone grafts already employed in the surgical routine as *Ricinus communis* (castor oil) polyurethane and autogenous bone marrow.

## Methods

### Animals

Forty-eight adult male New Zealand rabbits (*Oryctolagus cuniculus*), weighing 3.0 ± 0.5 kg, obtained from the Animal House of the State University of Maranhão (Universidade Estadual do Maranhão - UEMA) were used. The animals were kept at a temperature of 24 ± 1 °C and received ration and water ad libitum. The animals were acclimated for 10 days and handled under the same conditions. For the experiment, the rabbits were divided randomly into four groups: Control group, *C. ambrosioides* graft group, Autogenous bone marrow group and *Ricinus communis* graft (castor oil) group. All surgery was performed under ketamine hydrochloride and xylazine hydrochloride anesthesia, and all efforts were made to minimize suffering.

### Plant

*Chenopodium ambrosioides* leaves were collected at the time of use, in March 2009 from the “Berta Lanjes de Morretes” Medicinal Plant Garden, Federal University of Maranhão (São Luís, MA, Brazil). A voucher specimen was cataloged, identified and deposited under the registration number 0998.

### Preparation of gel from the lyophilized aqueous extract of *C. ambrosioides*

The aqueous extract was obtained by macerating fresh leaves (1000 g) in distilled water (1:4, *v*/v) at room temperature, under mechanical stirring for 24 h. Next, the aqueous extract was concentrated under reduced pressure and lyophilized (Fauber - LB1500, Terroni Equipamentos, São Carlos-SP), yielding 90 g and subsequently used in the preparation of *C. ambrosioides* gel for use as a graft [19] in experimental protocols. As a basis gel was used carbopol gel. Each tube of 20 g of gel obtained exhibited lyophilized aqueous extract of *C. ambrosioides* 5%, pH 6, dark green color and odor characteristic of the plant species.

### Phytochemical analysis

The methanol extract was subjected to phytochemical analysis for constituent identification using the phytochemical methods, which were previously described [20]. In general, tests for the presence or absence of phytochemical compounds were involved the addition of an appropriate chemical agent to the preparation in a test tube. The presence or absence of saponins, flavonoids, tannins, alkaloids was subsequently detected.

### Determination of DPPH·scavenging assay

The antioxidant activity of the extracts was measured on the basis of the scavenging activity of the stable 1, 1- diphenyl 2-picrylhyorazyl (DPPH) free radical according to the method described by Brand-Williams et al. [21] with slight modifications [21]. DPPH solution (1 ml/0.1 mM) in methanol was mixed with 1 ml of plant extract solution of varying concentrations (1,5, 3, 6, 12, 24, 48 and 100 μg/ml). The reaction was carried out in triplicate and the decrease in absorbance (A) was measured at 517 nm using UV-vis spectrophotometer. Gallic acid was used as a positive control. DPPH free radical scavenging ability (%) was calculated by using the formula:

$$ \left[{\mathrm{A}}_{517\ \mathrm{nm}\ \mathrm{of}\ \mathrm{control}}/{\mathrm{A}}_{517\ \mathrm{nm}\ \mathrm{of}\ \mathrm{sample}}\Big)/{\mathrm{A}}_{517\ \mathrm{nm}\ \mathrm{of}\ \mathrm{control}}\right]\ \mathrm{X}\ 100 $$

Results were expressed as IC50.

### Anesthesia and creation of bone defects

The animals were anesthetized by intramuscular injection of 5% ketamine hydrochloride (30 mg/kg) and 2% xylazine hydrochloride (4 mg/kg). An incision was made in the skin and subcutaneous tissue in the middle third of the left radius. A complete, simple, transverse diaphyseal fracture (1 cm) was then created at this site with an oscillating bone saw. In *C. ambrosioides* and *Ricinus communis* grafts, sufficient amounts of the bone grafts were applied to fill the bone defect. In Autogenous bone marrow, 1.0 ml of a bone marrow aspirate was collected from the iliac crest with a biopsy needle and injected directly into the fracture focus. The tissues were closed with simple sutures. During the postoperative period, dressings were applied daily for 10 consecutive days and the sutures were removed after this period. The animals were euthanized with lethal doses of the anesthetics used in the surgical procedure.

### Radiographical analysis

To monitor fracture consolidation and the formation of a bone callus, radiographs were obtained with a 100-mA X-ray apparatus (Siemens®, Hamburg-Germany) operating at 40 kV and 0.5 mA/s, using a 18 × 24 cm film. The radiographs were obtained in the craniocaudal and mediolateral positions 30, 60 and 90 days after surgery. For this analysis, the intensity of the periosteal reaction, closure of the fracture line, bone bridge, and bone callus formation were evaluated [22].

### Biochemical markers of bone formation

Blood samples were collected from the animals 30, 60 and 90 days after surgery for serum measurement of the following bone formation markers: osteocalcin were measured by electrochemiluminescent immunoassay by Elecsys 1010/2010 Modular analytics E170 analyser with the Roche Elecsys 1010/2010 (Roche Diagnostics, Mannheim, Germany), and thermostable bone alkaline phosphatase (BAP) measured with the Labtest® kit in a semi-automated apparatus - Bioplus® (Bioplus Produtos para Laboratório Ltda., Barueri, São Paulo).

### Biomechanical evaluation

Tension testing of the fractured segments was performed after 60 and 90 days to monitor the evolution of bone callus strength. A computerized universal testing machine (model TT2420, TIRA Maschinenbau, Germany) was used, applying a standard load in the velocity of 5 mm/min. The maximum load (force, N) and maximum deformation (stretching) were obtained from the result of the tensile force sustained by the specimens and is expressed as maximum force [23].

### Histological analysis

Histological analysis was performed 30, 60 and 90 days after surgery and application of the lyophilized aqueous *C. ambrosioides*, *Ricinus communis* and Autogenous bone marrow grafts to monitor the evolution of bone callus formation. The formation of new fibrous, cartilaginous and bone tissues during the healing process was analyzed. Cross-sections were removed from the fractured bone segments and fixed in 10% buffered formalin for 24 h [24]. After decalcification in 10% nitric acid, the fragments were processed and stained with hematoxylin-eosin and Sirius red [25, 26]. The histological sections were submitted to descriptive qualitative analysis to determine the pattern of bone regeneration.

### Statistical analysis

The results were expressed as the mean ± standard error of the mean (SEM) and were submitted to analysis of variance (ANOVA), followed by the Newman-Keuls post-test, using the GraphPad Prism 5.0 program. A *p* < 0.05 was considered significant. The histological results were reported qualitatively.

## Results

### Phytochemical screening and DPPH radical scavenging activity of *C. ambrosioides*

Phytochemical analysis of the aqueous extract of *C. ambrosioides* revealed the strong presence of flavonoids, alkaloids and saponins as shown in Table 1.

**Table 1:** Phytochemical screening of the aqueous extract of *Chenopodium ambrosioides* L.

Class of compounds	Aqueous extract
Flavonoids	+++
Alkaloids	++
Tannins	+
Phenolic compound	++
Catechins	+++
Saponins	+++
Anthocyanin	+++
Steroids	+
Terpenes	-

+: Presence of constituents; −: Traces of constituents; 0: Absence of constituents

Free radical scavenging activities of extract were assessed by the DPPH assay. The results show that aqueous extract of *C. ambrosioides* presented high DPPH elimination activity with a with an IC_50_ value of 39,10 ± 2,90 μg/ml. IC50 value of the positive control Gallic acid was 1,5 ± 0,35 μg/mL.

### Radiographic analysis of the *C. ambrosioides*, autogenous bone marrow and *Ricinus communis* grafts

**Fig. 1:**
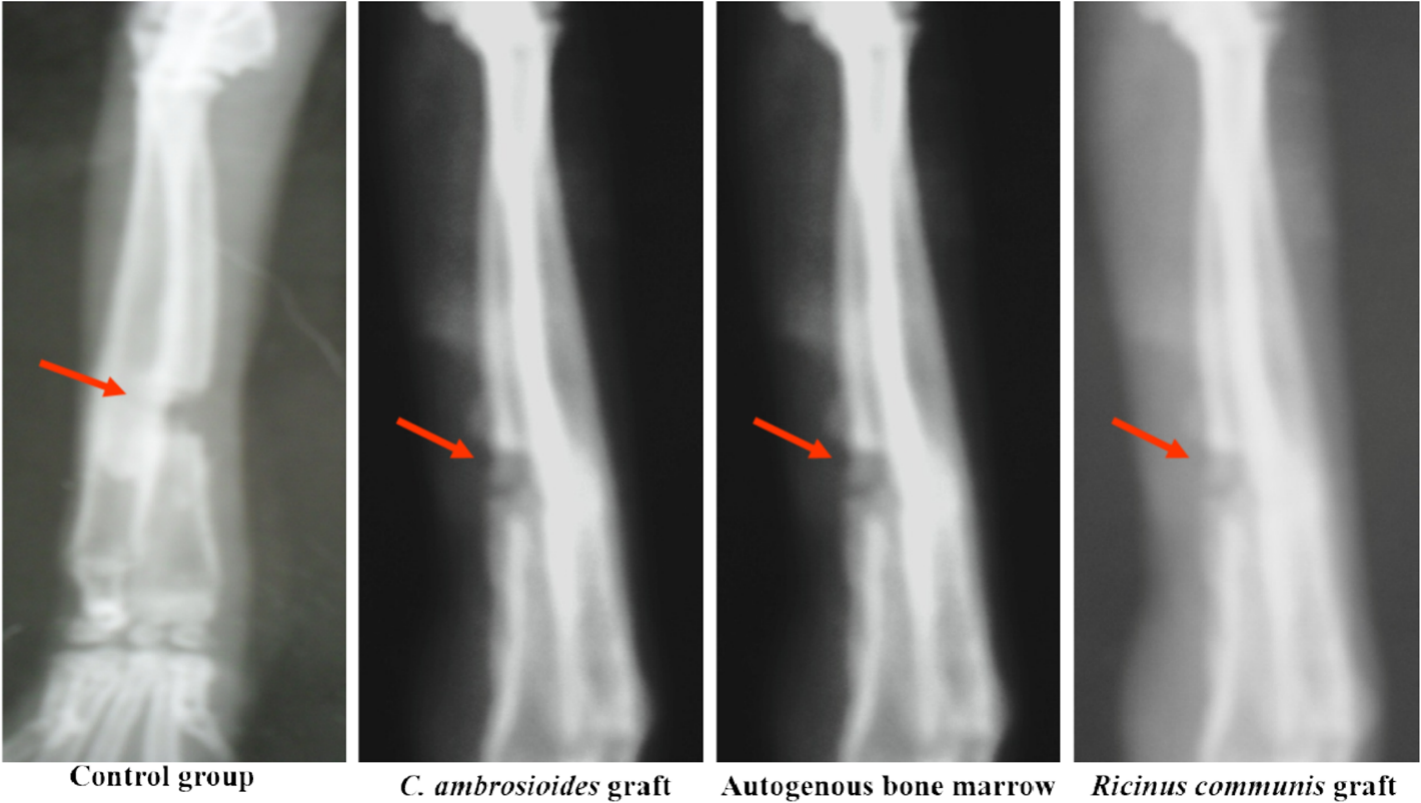
Effect of a graft of lyophilized aqueous *Chenopodium ambrosioides* L. extract on bone formation. Radiographs showing bone callus evolution after 30 day. Control group and *Ricinus communis* graft bone defect exhibiting the absence of a fibrous bone callus. *C. ambroioides* graft and Autogenous bone marrow bone defect exhibiting the beginning of formation of a fibrous bone callus

Grafted material was observed in the fracture focus in all groups at 30 days, as well as the presence of a fracture line and a mild periosteal reaction. The onset of a periosteal reaction was only seen in *C. ambrosioides* graft and Autogenous bone marrow (Fig. 1).

**Fig. 2:**
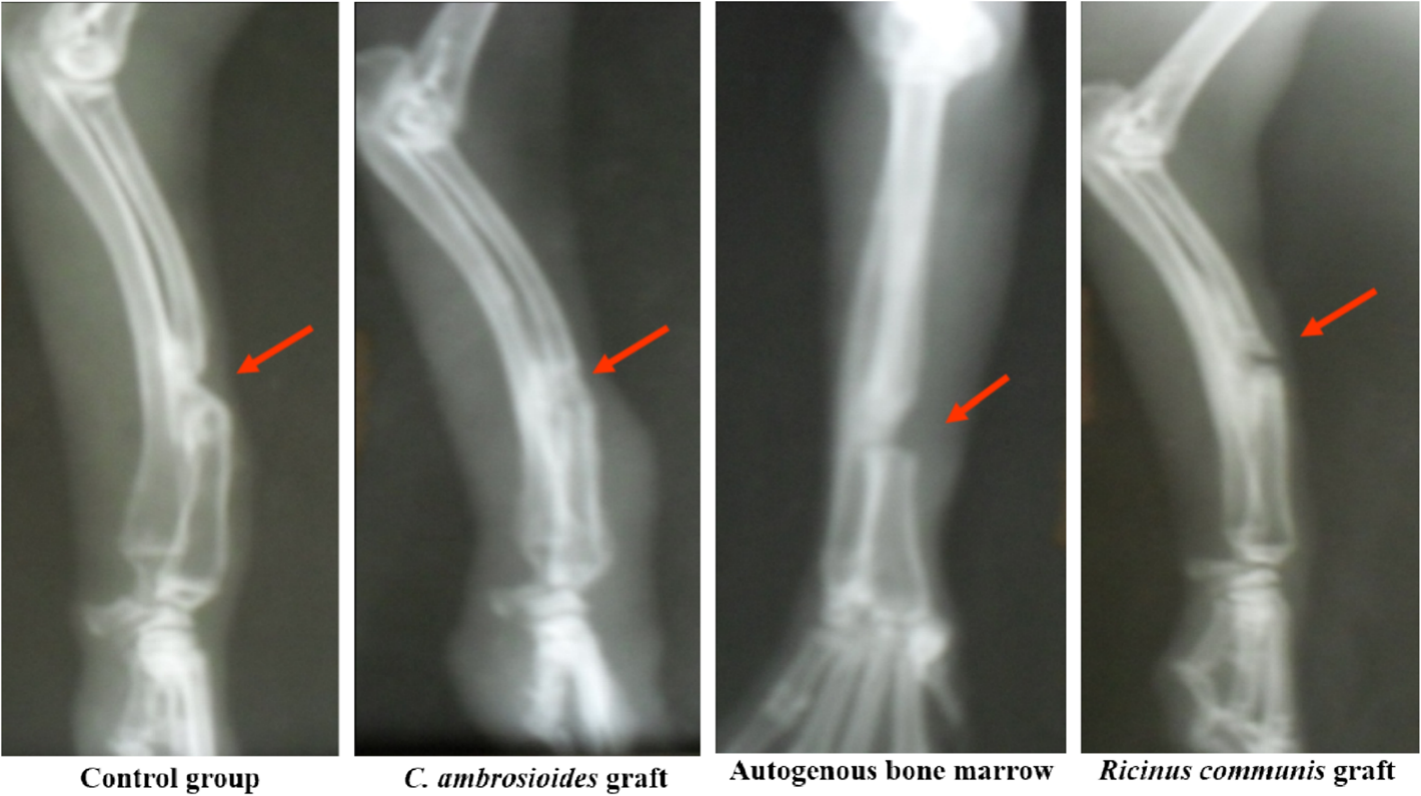
Effect of a graft of lyophilized aqueous *Chenopodium ambrosioides* L. extract on bone formation. Radiographs showing bone callus evolution after 60 day. Control group: Bone defect exhibiting the formation of a fibrous bone callus. *C. ambroioides* graft: Formation of a fibrous bone callus throughout the fracture line. Autogenous bone marrow: Bone defect exhibiting the beginning of formation of a fibrous bone callus. *Ricinus communis* graft: Bone defect exhibiting the formation of a fibrous bone callus and retraction of the fracture line

At 60 days, a fibrous bone callus started to form in all groups, but the fracture line was absent only in *C. ambroioides* graft (Fig. 2).

**Fig. 3:**
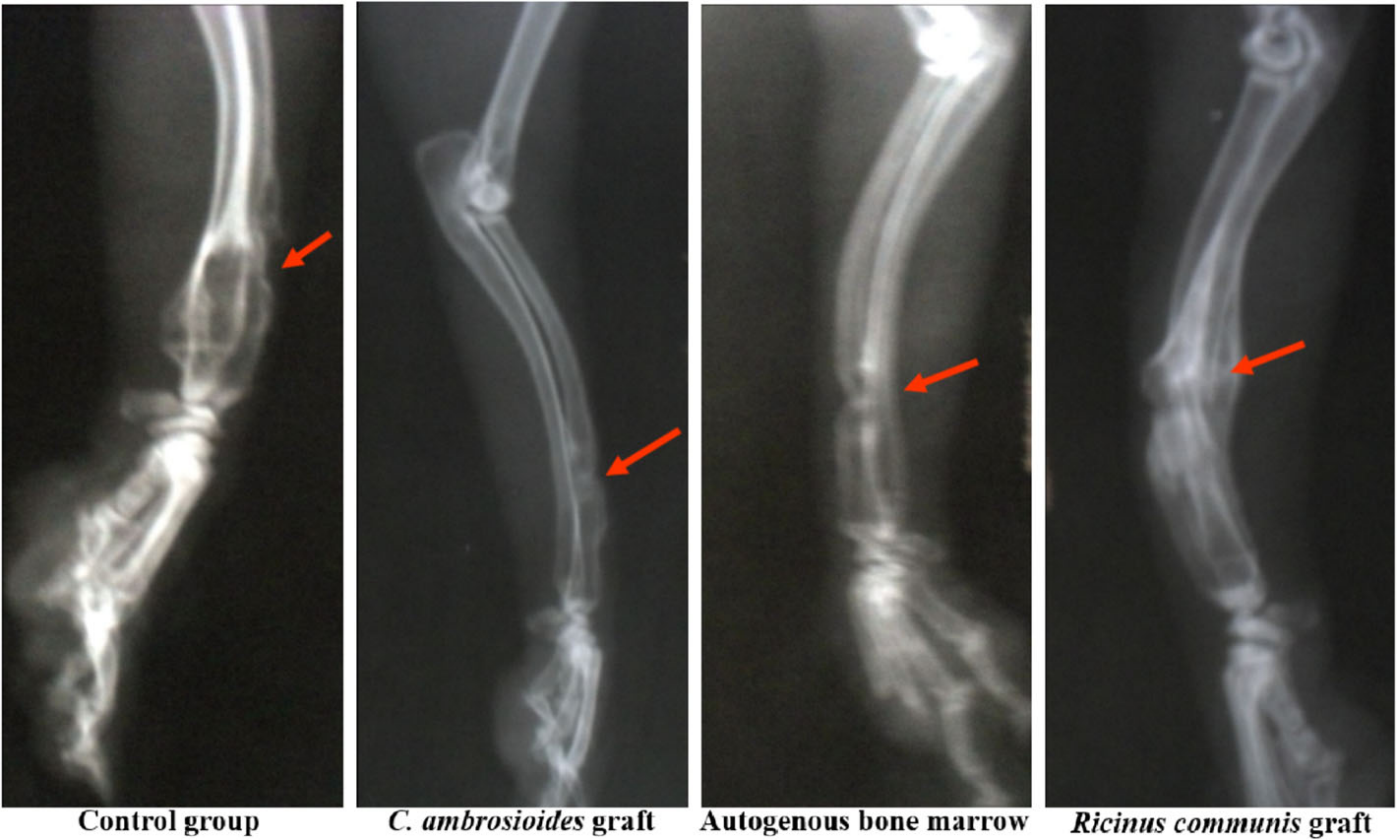
Effect of a graft of lyophilized aqueous *Chenopodium ambrosioides* L. extract on bone formation. Radiographs showing bone callus evolution after 90 day. Groups: Control group, *C. ambrosioides* graft, Autogenous bone marrow and *Ricinus communis* graft. Control group: Formation of a prominent fibrous bone callus. *C. ambrosioides* graft: Formation of a mature bone callus throughout the fracture line. Autogenous bone marrow: Formation of a mature bone callus. R*icinus communis* graft: Formation of a prominent fibrous bone callus

After 90 days, consolidated fractures were already observed in *C. ambroioides* graft and Autogenous bone marrow, but a cortical fracture line was still present in the latter. In contrast, a mature bone callus with an exuberant periosteal reaction was observed in the Control group and *Ricinus communis* graft (Fig. 3).

### Biochemical markers of bone formation of the *C. ambrosioides*, autogenous bone marrow and *Ricinus communis* grafts

After application of the grafts, only *C. ambrosioides* graft presented an increase in BAP at 30 (54.06 ± 2.80% and 43.50 ± 1.70%, respectively) and 60 days (49.07 ± 5.51% and 25.64 ± 6.40%, respectively) when compared to the Control group (Table 2). A significant difference in osteocalcin was observed between *C. ambrosioides* graft and the Control group at 30 days (9.0 ± 1.3 and 4.05 ± 1.0 ng/ml, respectively) (Table 2).

**Table 2:** Comparison of bone alkaline phosphatase and osteocalcin after the application of *Chenopodium ambrosioides* L.

Group	Bone Alkaline Phosphatase (%)			Osteocalcin (ng/ml)		
	30 days	60 days	90 days	30 days	60 days	90 days
Control	43.5 ± 1.7	25.6 ± 6.4	45.4 ± 2.4	4.0 ± 1.0	10.1 ± 0.8	8.6 ± 1.0
*C. ambrosioides* graft	54.1 ± 2.8*	49.1 ± 5.5*	41.1 ± 4.8	9.0 ± 1.3*	13.8 ± 1.7	9.4 ± 1.9
Autogenous bone marrow	44.4 ± 2.7	32.4 ± 4.4	38.1 ± 6.9	7.2 ± 1.0	18.0 ± 1.9*^,a^	14.3 ± 1.8
*Ricinus communis* graft	45.9 ± 3.7	29.8 ± 0.6	37.4 ± 5.1	5.9 ± 2.1	8. 5 ± 2.2	10.0 ± 1.7

Results are reported as the mean ± SEM. *p < 0.05, significantly different compared to Control group. ^a^Significant difference compared to the *Ricinus communis* grafts (ANOVA, Tukey test)

### Biomechanical assessment of the *C. ambrosioides*, autogenous bone marrow and *Ricinus communis* grafts

**Fig. 4:**
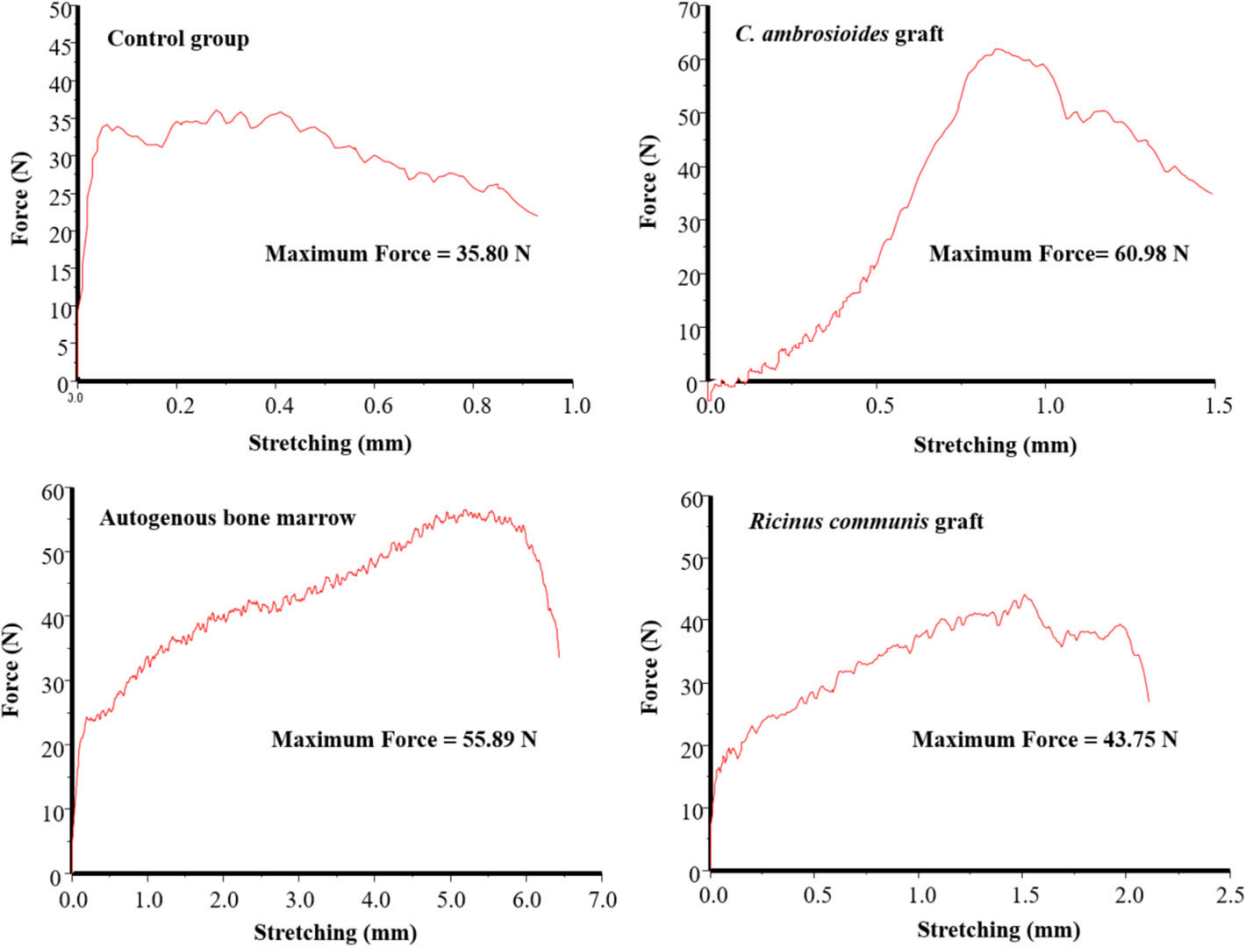
Effect of a graft of lyophilized aqueous *Chenopodium ambrosioides* L. extract on bone formation. Biomechanical analysis at 60 days. The results are reported as maximum force (Fmax). Groups: Control group, *C. ambrosioides* graft, Autogenous bone marrow e *Ricinus communis* graft

At 60 days, applying a load velocity of 5 mm/min, specimens of *C. ambrosioides* graft and autogenous bone marrow withstood the same maximum force, with the observation of a slightly higher tensile strength in 60.98 and 55.89 N, respectively (Fig. 4).

**Fig. 5:**
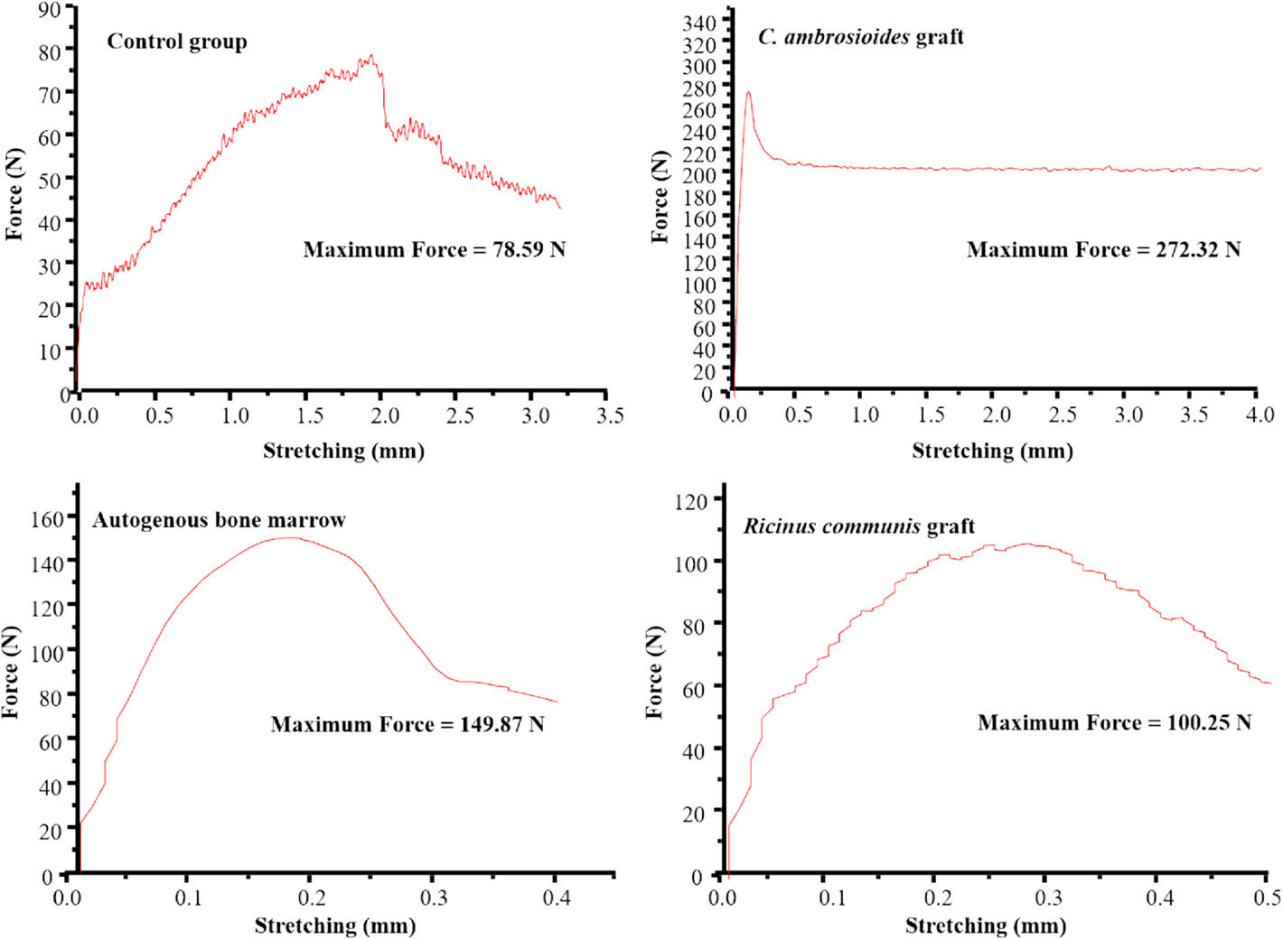
Effect of a graft of lyophilized aqueous *Chenopodium ambrosioides* L. extract on bone formation. Biomechanical analysis at 90 days. The results are reported as maximum force (Fmax). Groups: Control group, *C. ambrosioides* graft, Autogenous bone marrow e *Ricinus communis* graft

At 90 days, specimens of *C. ambrosioides* graft withstood approximately double the maximum force compared to Autogenous bone marrow (272.32 and 149.87 N, respectively) when the same velocity was applied (Fig. 5).

### Histological analysis of the *C. ambrosioides*, autogenous bone marrow and *Ricinus communis* grafts

**Fig. 6:**
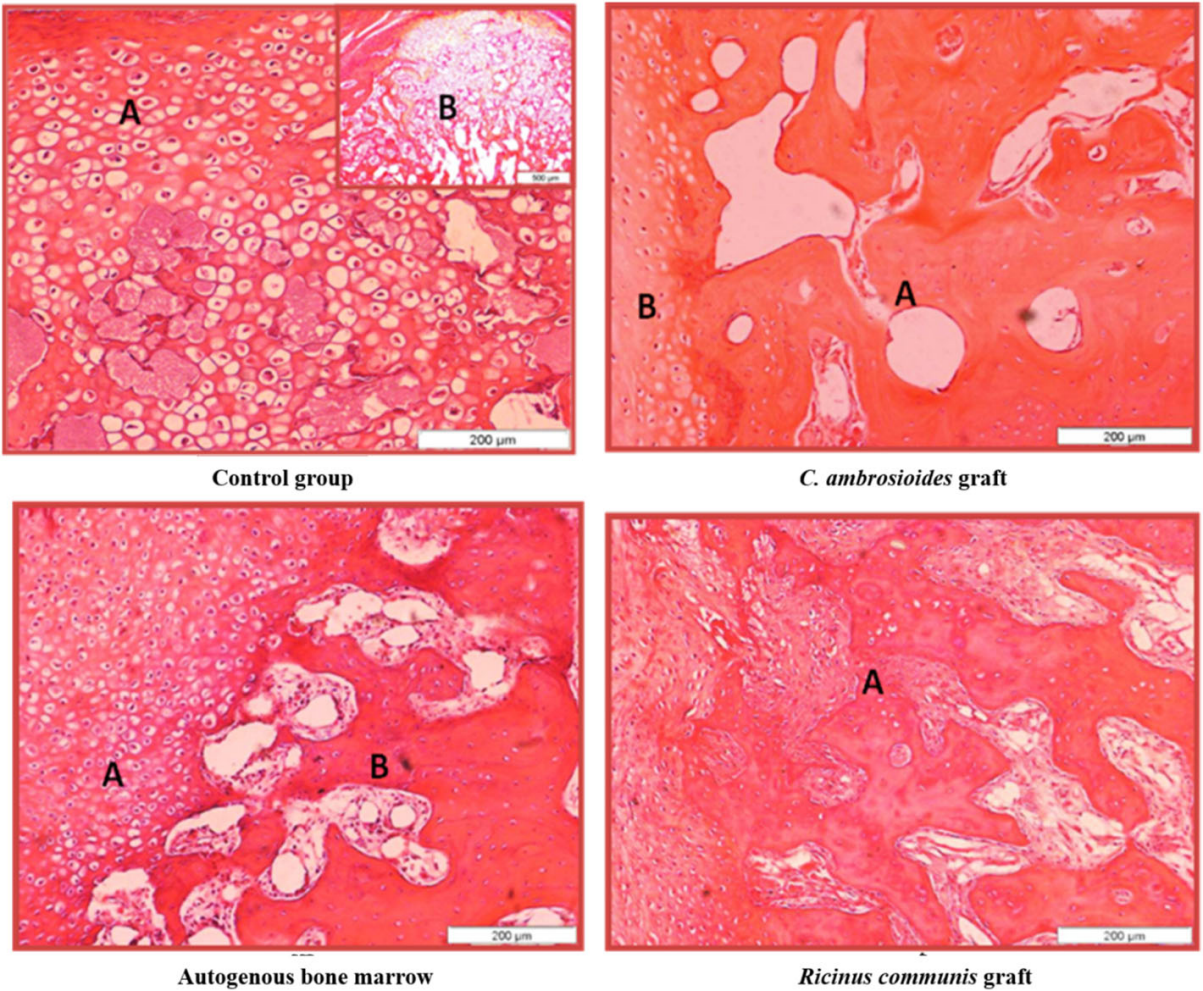
Effect of lyophilized aqueous *Chenopodium ambrosioides* extract on bone formation 30 days after application. Histology photomicrographs of bone remodeling in a complete diaphyseal fracture of the radius of rabbits. Control group: (**a**) massive presence of chondrocytes and (**b**) predominance of cartilaginous tissue. *C. ambrosioides* graft: (**a**) presence of immature bone and (**b**) cartilaginous tissue. Autogenous bone marrow: (**a**) cartilaginous tissue and (**b**) immature bone. *Ricinus communis* graft: (**a**) chondrocytes and bone remodeling. HE staining (200 μm and 500 μm)

Thirty days after application of the grafts (Fig. 6), a predominance of immature bone and discrete presence of cartilage were only observed in *C. ambrosioides* graft animals. On the other hand, abundant cartilaginous tissue (cartilaginous collar) was seen in Control animals which were also submitted to complete diaphyseal fracture of the radius, but were not treated with the grafts. A large number of chondrocytes and the onset of bone remodeling were observed in *Ricinus communis* graft, whereas immature bone (non-lamellar bone) and the massive presence of cartilaginous tissue were noted in Autogenous bone marrow. At 60 days, all groups exhibited similar tissue architecture, including the presence of endochondral ossification and osteoclasts, and mature bone was observed at 90 days.

**Fig. 7:**
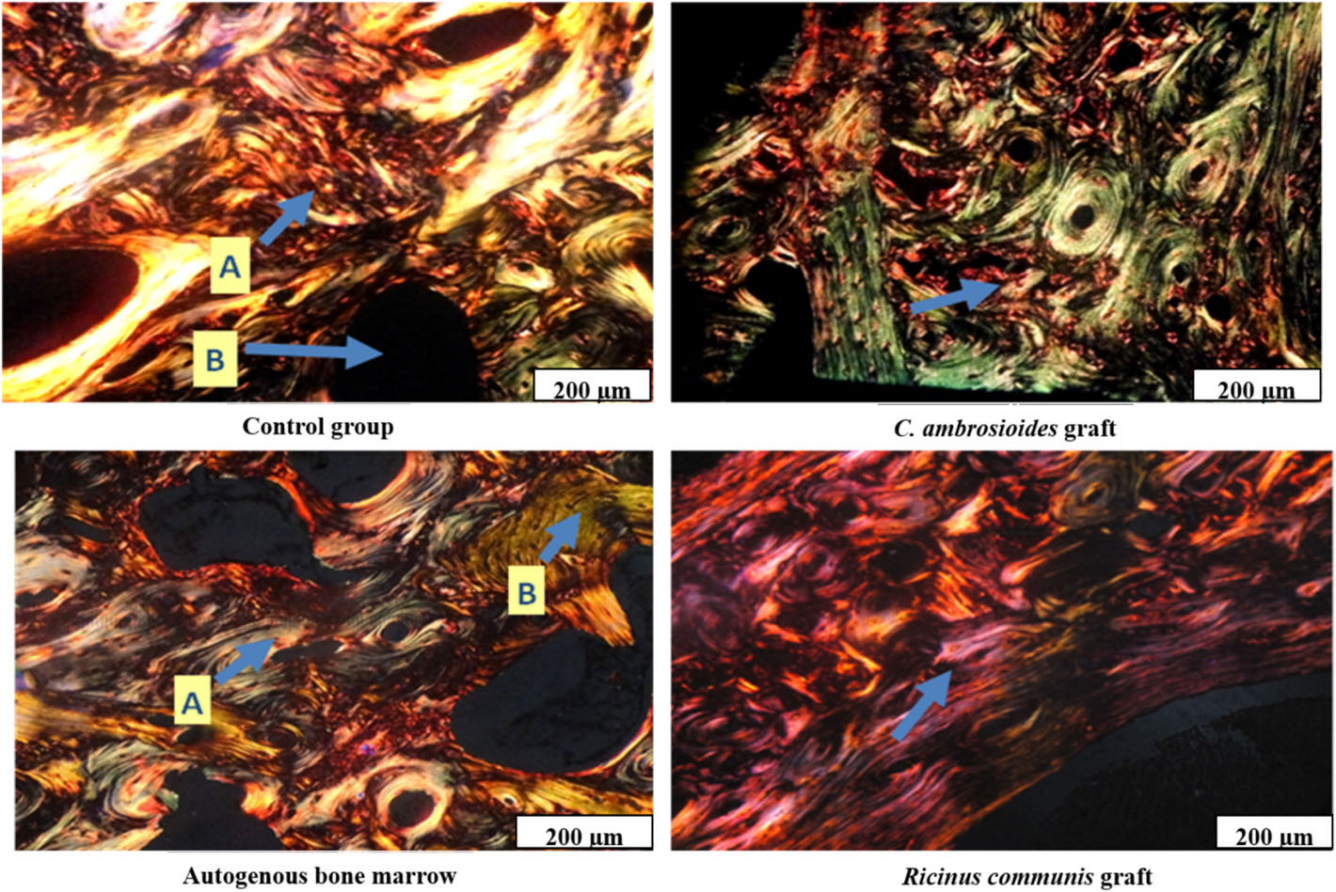
Effect of lyophilized aqueous *Chenopodium ambrosioides* extract on bone formation 30 days after application. Histology photomicrographs of collagen organization of bone remodeling in a complete diaphyseal fracture of the radius of rabbits. Control group: (**a**) non-organized collagen and (**b**) beginning of osteon formation. *C. ambrosioides* graft: mature osteon. Autogenous bone marrow: predominance of non-lamellar bone tissue. *Ricinus communis* graft: (**a**) predominance of a forming osteon and (**b**) non-organized collagen. Sirius red staining under polarized light (200 μm)

Analysis of collagen at 30 days (Fig. 7) showed the formation of a mature osteon and greater collagen organization in *C. ambrosioides* graft when compared to the Control group. A forming osteon predominated in Autogenous bone marrow and non-lamellar bone in *Ricinus communis* graft. Both groups exhibited less organized collagen when compared to *C. ambrosioides*.

## Discussion

We evaluated the bone repair process in rabbit radio with significant loss of osseous tissue and use of natural osseous graft as a gel of lyophilized aqueous extract of *Chenopodium ambrosioides*. The findings indicate that the gel of the plant promotes early bone formation by stimulating osteoblast production increasing tissue resistance, and favoring better collagen deposition, being biocompatible this way rabbits.

In the process of bone repair occurs equilibrium between deposition and tissue absorption, the assessment of specific biomarkers of new bone tissue formation is required. Bone alkaline phosphatase, the bone-specific isoform of alkaline phosphatase, is a specific marker of bone formation, is only produced by osteoblasts and is essential for bone mineralization [27]. Elevated levels of bone alkaline phosphatase are observed during the process of fracture consolidation [28]. In this respect, Peng et al. [29], studying the effect of a topical paste consisting of the extracts of different herbs on bone repair in rabbits, observed an increase in bone alkaline phosphatase activities [29]. In the present study, an increase in the serum levels of this enzyme was seen in animals receiving the *C. ambrosioides* graft 30 and 60 days after fracture creation (Table 2), revealing that this medicinal plant can stimulate bone alkaline phosphatase activity during early healing of the fracture in a time dependent manner, suggesting osteogenesis.

Another specific marker of bone formation is osteocalcin, which accounts for 80% of the gamma-carboxyglutamyl content of mature bone. Osteocalcin is important for the diagnosis and investigation of bone alterations and serves as an indicator of the formation and maturation of bone tissue [28, 30]. In the present study, no significant difference in osteocalcin was observed between *C. ambrosioides* graft and the other groups after 30, 60 and 90 days of bone callus formation (Table 2). In contrast, animals that received Autogenous bone marrow showed elevated serum levels of osteocalcin at 60 days compared with the control group, suggesting this stage more active bone metabolism.

Radiographic analysis at 30 days revealed the formation of bone callus in the fibrous *C. ambrosioides* graft demonstrating early bone regeneration (Fig. 1), similarly to Autogenous bone marrow. Previous results reported in literature corroborate our results by Lima et al. [31] who used bone morphogenetic proteins for the consolidation of radius fractures in rabbits [31], and by Freitas et al. [32] who observed bone mineralization after application of a cortical bone heterograft in the same species [32].

In addition, the results show a fracture line absent only in *C. ambrosioides* graft animals after 60 days, indicating early bone formation (Fig. 2), in contrast to other groups where there was still prominent bone defect. These findings contrast with those reported by Guerra et al. [33], in the study of bone perforations in dogs, still detected the presence of a fracture line at 60 days, but in a stage of consolidation [33]. The formation of the mature bone callus was observed in all groups at 90 days (Fig. 3), agreeing with the findings of Pinheiro Neto et al. [8], which evaluated the action of *C. ambrosioides* poultice of leaves in osseous tissue of rabbits [8].

The results of the tensile strength test showed a better performance in *C. ambrosioides* graft at 60 days (Fig. 4) and 90 days (Fig. 5), compared to other groups suggesting more mature consolidation and increased resistance of bone tissue and confirming the radiographic results. Biomechanical assays should only be performed after 60 days in view of the evolution of bone callus formation, as reported by Peng et al. [29] using a paste of herbs for fracture treatment in rabbits [30] and by Kupczik et al. [34] using ciprofloxacin for bone consolidation of rat femoral fractures [34].

In continuity, significant histological alterations were only observed after 30 days, when *C. ambrosioides* graft animals exhibited lamellar bone (Fig. 6) and better collagen organization (Fig. 7) compared to the other groups. The tissue architecture was similar in all groups at 60 and 90 days, with the observation of the presence of endochondral ossification, osteoclasts and mature bone. Wong and Rabie, 2006, 2007, 2010, when analyzed different plant species on bone formation also observed similar results [35–37]. Furthermore, Pereira Junior et al., [38] also report a comparative study between polyurethanes containing castor oil (soft segment) and cancellous bone autograft in the treatment of segmental bone defects induced in rabbits [38]. As well by Laureano Filho et al., [39] analyzed the effect of *Ricinus communis* polymer on bone regeneration in rabbit calvaria [39].

Flavonoids are the most ubiquitous groups of plant secondary metabolites [40], comprising flavones, isoflavones and neoflavonoides and have, as one of the main functions, protection against oxidative stress in cells [41, 42]. This class has direct effects on bone metabolism, such as decreased bone resorption by osteoclasts, promotion of differentiation and pro-osteoblastic cell mineralization, and an increase in alkaline phosphatase activity [43]. Thus, the presence of flavonoids in *C. ambrosioides* graft may be contributing to its effect on bone neoformation.

DPPH radical scavenging is considered a good in vitro model widely used to assess antioxidant efficacy within a very short time [44]. In its radical form, DPPH· has disappears on reduction by an antioxidant compound or a radical species to become a stable diamagnetic molecule resulting the colour change from purple to yellow, which could be taken as an indication of the hydrogen donating ability of the tested sample [45, 46].

The results of this study revealed the removal capacity of DPPH of *C. ambrosioides*, but lower than that of gallic acid. On the other hand, however, its value is appreciable and antioxidant activity can be attributed. This finding is in agreement with other studies reported in the literature for antioxidant activity of *C. ambroisoides* [47, 48]. Removal of free radicals is positively associated with increased differentiation and proliferation of osteoblasts [49], which may explain the effects of *C. ambroisoides* graft on early bone healing.

## Conclusion

In conclusion, the findings of this study suggest that the *C. ambrosioides* graft proved to be biocompatible bone by inducing earlier bone neoformation. Further studies are being developed in our laboratory in order to evaluate the clinical efficacy of this gel formulation as bone graft.
